# Personalised community based telerehabilitation post stroke to increase rehabilitation time and improve motor recovery (PRACTISE): study protocol for a randomised controlled feasibility trial

**DOI:** 10.1186/s40814-026-01897-5

**Published:** 2026-08-19

**Authors:** Colin B. Shore, Elaine H. Coulter, Lesley Scobbie, Lisa Kidd, Frederike Van Wijck, Carol Bugge, Helen Mason, Nicole Irvine, Mark Barber, Katie I Gallacher, Jacqui Morris, Graeme MacLennan, Pete White, Keith Jenkinson, Lorna Paul

**Affiliations:** 1 School of Health and Life Sciences, Glasgow Caledonian University, Glasgow, United Kingdom; 2 NHS Lanarkshire, Bothwell, United Kingdom; 3 Yunus Centre for Social Business and Health, Glasgow Caledonian University, Glasgow, United Kingdom; 4 School of Health and Wellbeing, University of Glasgow, Glasgow, United Kingdom; 5 School of Health Sciences, University of Dundee, Dundee, United Kingdom; 6 Centre for Healthcare Randomised Trials, Aberdeen Centre for Evaluation, University of Aberdeen, Aberdeen, United Kingdom; 7 Patient & Public Partnership Group to PRACTISE, Glasgow Caledonian University, Glasgow, United Kingdom

**Keywords:** Stroke, Physiotherapy, Exercise, Telerehabilitation, Feasibility, Randomised controlled trial, Process evaluation, Rehabilitation

## Abstract

**Introduction:**

Stroke is the second leading non-communicable cause of death and third leading cause of death and disability worldwide with prevalence expected to rise 120% between 2015 and 2035. The first 6 months after stroke is the optimum time for neuroplasticity and a critical period for rehabilitation. UK national clinical guidelines recommend stroke survivors should receive at least 3 h/day of multidisciplinary rehabilitation at least 5 days/week. Workforce shortages and service variability make achieving this standard consistently challenging. Goal setting, telerehabilitation, and self-management strategies are advocated, yet their combined use in community-based rehabilitation has not been tested. This study aims to conduct a randomised feasibility-controlled trial with embedded process evaluation to assess the feasibility of delivering a 16-week, personalised, home-based physiotherapy, prior to progression to a full-scale trial.

**Methods and analysis:**

This trial will recruit 60 participants with moderate post-stroke disability across Scotland. Participants will be randomised to the PRACTISE intervention or control (usual physiotherapy). PRACTISE (Personalised community-based telerehabilitation post stroke to increase rehabilitation time and improve motor recovery) is a 16-week, personalised physiotherapy programme involving goal setting, supported self-management and exercise via an online video-based platform. PRACTISE consists of five home-based physiotherapy sessions and four online/telephone sessions with participants encouraged to engage in a personalised exercise programme up to five times/week for up to 3 h/day. Both groups will receive other usual stroke care.

Feasibility outcomes will include recruitment, retention, and completion rates. A process evaluation will assess acceptability and implementation factors. Clinical outcomes, recorded at weeks 0, 8, 16, and 24 by a blinded assessor, include the Canadian Occupational Performance Measure (primary outcome) and measures of limb function, fatigue, quality of life, functional ability, treatment burden, falls, self-efficacy, caregiver strain, and healthcare resource use (secondary outcomes). Exercise dosage and adherence will be monitored via exercise diaries.

**Discussion:**

Intensive post-stroke rehabilitation is recommended but challenging to achieve. PRACTISE combines goal setting, self-management, and online exercise prescription to support stroke survivors to achieve recommended rehabilitation doses. This study will examine the feasibility, acceptability, and mechanisms of the intervention to inform potential refinement and progression to a full-scale trial.

**Trial registration:**

Prospectively registered at https://clinicaltrials.gov. (NCT06871878).

## Introduction

Globally, one in four people above 25 years will have a stroke in their lifetime [1]. Stroke remains the second leading cause of death [1] and affects around 100,000 people each year in the UK, with its prevalence predicted to increase by 120% between 2015 and 2035 [2]. The first 6 months after stroke is the optimum period to promote neuroplasticity, where the brain has the ability to repair and reorganise in response to new experiences and stimuli [3]. As such, this is a critical period for rehabilitation, which involves repetition of tasks to induce lasting changes [3–6]. The type of exercise post-stroke needs to be task-specific, and the dose must be adequate to drive neuroplasticity and thereby optimise motor and functional recovery [7, 8]. However, limited rehabilitation time and limited opportunities for engaging in rehabilitation of sufficient intensity [9] can negatively impact neuroplastic changes [6].

The 2023 UK national clinical guideline for stroke recommends stroke survivors should receive at least 3 h a day of multidisciplinary rehabilitation at least 5 days a week (National Institute for Health and Care Excellence [10, 11]. This brings the UK in line with similar recommendations in different countries including USA, Australia and New Zealand [12, 13]. Delivering this dose of rehabilitation in community settings is challenging due to multiple factors including increasing demands on stroke services and chronic workforce shortages ([14]. Around 40% of stroke survivors are discharged from hospital without any further rehabilitation [15]. Furthermore, those are seen are not likely to be seen by rehabilitation staff at a level that matches current recommendations. Subsequently, stroke survivors may receive sub-optimal doses of therapeutic rehabilitation. Therefore, alternative models of community rehabilitation are needed.

Goal setting and supported self-management are fundamental components of stroke rehabilitation practise recommended in clinical guidelines (National Institute for Health and Care Excellence [10, 11]. Evidence demonstrates the importance of person-centred goal setting and supported self-management on rehabilitation outcomes, self-efficacy and quality of life [16–18]. Person-centred approaches to goal setting and supported self-management position the patient as working in active collaboration with clinicians in the planning of their care and rehabilitation [19]. Crucially, however, confidence for achieving goals and being able to self-manage comes from feeling supported by professionals in a trusted therapeutic relationship and not just being left to get on with it alone [18, 20, 21].

Telerehabilitation has the potential to address challenges in delivering intensive rehabilitation across geographical contexts [22]. Telerehabilitation in the context of stroke is a rapidly growing area of interest and evidence shows that it is comparable to in-person rehabilitation across multiple outcomes [23–25]. National guidelines in the UK, USA, Australia and New Zealand recommend telerehabilitation to overcome barriers of access to services, or as an adjunct to in-person rehabilitation (National Institute for Health and Care Excellence [10–13].

The aim of this study is to conduct a randomised controlled trial (RCT) with an embedded process evaluation to assess the feasibility of delivering a 16-week, community-based physiotherapy programme, incorporating personalised goal setting, supported self-management and telerehabilitation, designed to increase rehabilitation exercise time among stroke survivors. Specifically, looking to increase meaningful rehabilitation exercise undertaken by patients beyond direct clinical therapy time.

## Methods and analysis

### Design and aim

This is a pragmatic, multicentre, single-blinded, feasibility RCT with embedded process evaluation. The aim is to assess the feasibility of delivering the intervention as intended, with feasibility assessed using the pre-specified progression criteria relating to consent, recruitment, intervention attendance, adherence, data completeness and intervention fidelity; identify barriers and facilitators to delivery; and offer preliminary insights into its potential to engage participants in their rehabilitation, improve functional outcomes and reduce disability, thereby informing the design of a larger definitive trial.

### Recruitment

Stroke survivors, caregivers or family members, and Physiotherapists involved in delivering the PRACTISE intervention, will be recruited from four NHS Health Boards in Scotland. These geographical areas will offer opportunities to explore the intervention within urban and rural communities and across socioeconomic diversity.

### Participants and eligibility criteria

Three different groups of participants will be recruited: stroke survivors, informal carers or family members, and physiotherapists, each with specific inclusion and exclusion criteria (Table 1). This study aims to recruit 60 participants who have experienced a stroke. Outcome data on 60 participants are sufficient to estimate the standard deviation required for a sample size calculation for a definitive trial [26]. Thirty participants will be randomised to the PRACTISE intervention, and 30 participants to the control group. Where appropriate, if stroke survivors have informal carers or family members, they will be invited to take part (up to 60 informal carers or family members). A subset of stroke survivors in each group (*n* = 14) and informal carers or family members (*n* = 14),physiotherapists (*n* = 6) will be interviewed.

**Table 1:** Inclusion and exclusion criteria of PRACTISE study participants

Participant group	Inclusion criteria	Exclusion criteria
Stroke survivors	• Over 16 years old • Within 3 months of first stroke • Requiring community physiotherapy and discharged home from in-patient care • Scores 2–4 on the modified Rankin Scale on discharge • Able to use a computer/tablet or mobile phone with/without help • Able to understand/communicate in English • Able to provide informed consent to take part • Able to provide informed consent for carer or family members to be approached	• Previous neurological disability • Participating in another physical interventional research study or inclusion in another study that would prevent participation in this study • Discharged to a nursing home or other long-term care facility or end of life care • Absolute contra-indication to exercise (e.g. acute myocardial infarction (within 2 days) or unstable angina, uncontrolled cardiac arrhythmias causing symptoms or haemodynamic compromise, acute pulmonary embolus or pulmonary infarction, acute myocarditis or pericarditis)
Informal carer or family member	• Informal carers or family members of an individual who is part of the PRACTISE study either as a control or intervention participant • An individual that lives with the stroke survivor, or sees them regularly in any given week • Over 16 years old • Able to provide informed consent to take part • Able to join and contribute to an interview, either in-person or via a video conference platform, in English	
Physiotherapist	• Physiotherapist who will deliver the PRACTISE intervention • Able to join and contribute to an interview via video conference platform or in person	

#### Stroke survivors

Individuals who have experienced a first stroke and are currently receiving inpatient care for this stroke will be screened by clinical staff (e.g. Research Nurse or Physiotherapist) for eligibility to the study (Table 1). Those eligible will be approached by a Research Nurse, member of the clinical team (e.g. Physiotherapist) or members of the study team (e.g. study co-ordinator) in the inpatient setting and asked if they would like to take part in the PRACTISE study. Interested individuals will be given a Participant Information Sheet (PIS) and have the opportunity to ask any questions. Stroke survivors will be given at least 48 h to read the PIS and to consider their participation. After time to consider, participants who wish to take part in the study will then be asked to provide written informed consent. If participants have capacity to give verbal consent, but lack physical capability to sign consent forms, a witness (e.g. family member) can provide written consent. Written informed consent will be taken prior to the participant being discharged home from hospital to receive their community physiotherapy.

#### Informal carers or family members

Stroke survivors will be made aware of the option, for informal carers or family members to be involved and asked to nominate a relevant carer or family member to be approached. It will be made clear that carer or family member involvement is not a requirement of their involvement in the study.

Recruitment of informal carers or family members may occur in the inpatient or community settings. The carer or family member will be provided with a PIS and given opportunity to ask questions. Participants will be given at least 48 h to read the PIS prior to providing informed consent.

#### Physiotherapists

In discussion with team leads, physiotherapists will volunteer themselves to be trained (described below) as a PRACTISE therapist. PRACTISE Physiotherapists will be provided with a PIS, the opportunity to ask questions, and have at least 48 h to consider their participation prior to consenting to the study. This includes consent to have a sub-sample of sessions audio-recorded and to be potentially interviewed as part of the embedded process evaluation.

#### Process evaluation interviews

At the time of taking consent to the intervention stage of the study, stroke survivor and informal carers or family members will also be asked for their permission to be approached to take part in a semi-structured interview as part of the embedded process evaluation (described below). Those who agree to be approached for the process evaluation will be contacted by a member of the research team towards the end of the 16-week study period. The research team member will explain the interview process, answer any questions, and allow 48 h of consideration before taking further informed consent for the process evaluation interview.

### Randomisation

Stroke survivors will be randomised either to control or intervention via a web-based system created and managed by the Centre for Healthcare Randomised Trials (CHART), University of Aberdeen. Randomisation will be 1:1 to intervention and control using randomly permuted blocks of size 2 and 4, stratified by NHS board. A named physiotherapist within the Health Board will conduct the randomisation and be informed of the group allocation of the participant via the web-based system. They will inform the appropriate team members to facilitate appointments. The study co-ordinator will also be notified of group allocation.

### Intervention

#### Intervention group

**Fig. 1:**
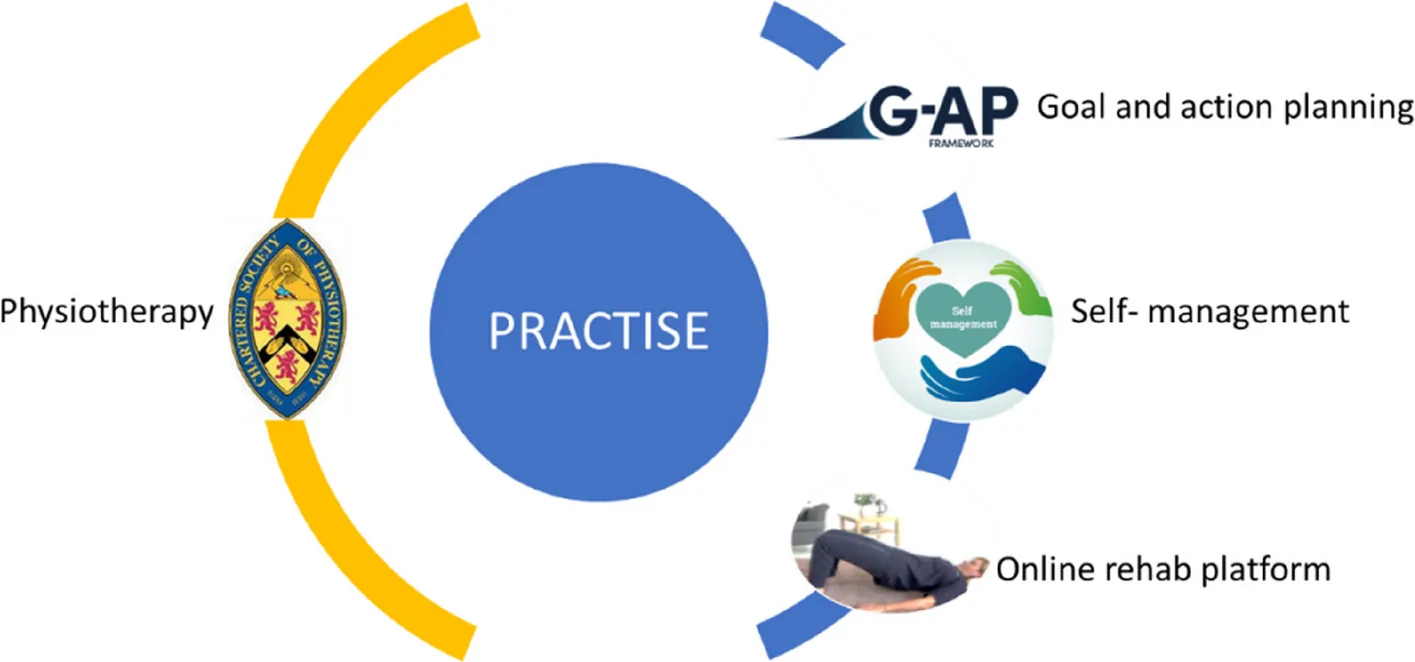
Overview of the components of the PRACTISE intervention

Stroke survivors randomised to the intervention group will receive the PRACTISE intervention, a 16-week personalised community-based physiotherapy programme, delivered via in-person and remote appointments (Fig. 1). This will include (1) usual physiotherapy assessment and treatment; (2) evidence-based goal setting and action planning (G-AP) [17],(3) a personalised, prescribed exercise programme delivered via an online exercise programme platform (https://www.giraffehealth.com); with an overarching (4) supported self-management approach [20]. Subsequently, we will seek to report the results and develop a framework for the future definitive study via a Template for Intervention Description and Replication (TiDieR).

#### Goal setting and action planning framework (G-AP)

The goal setting and action planning (G-AP) framework is a theory- and evidence-based method developed to support stroke survivors to consider, plan, and work towards their goals collaboratively with rehabilitation staff [17, 18]. Evaluations of G-AP in community stroke rehabilitation settings found it was acceptable to staff and stroke survivors, feasible to implement in practise and helpful in the setting and pursuit of person-centred goals [27, 28].

#### Online rehabilitation platform (Giraffe)

The Giraffe platform has been co-developed with patients and is an evidence-based, telerehabilitation platform. The platform allows therapists to provide personalised, video-based exercise programmes and with an inbuilt exercise diary to allow recording of exercise progression. The platform will be used by therapists to prescribe their rehabilitation exercises. Furthermore, it will support participants to exercise out with their therapy appointments. Previous research of the platform during in-patient stroke rehabilitation found it to be feasible, safe and acceptable to patients, carers and physiotherapists [29, 30].

#### Supported self-management

Supported self-management is an evidence-based approach ([31] that underpins approaches to goal setting and long-term support, like the G-AP framework. It is an approach that supports and guides practitioners on *how* to engage stroke survivors in collaborative discussions to help them to identify their self-management priorities and strategies and interventions that can help them to build their confidence and skills for living well, integrating their rehabilitation into their daily lives and for dealing with barriers or challenges along the way [32–34]. Self-management, and self-managed practise (engaging specifically in rehabilitation in the absence of therapist supervision), is seen as essential to maximise the dose of therapy and sustain treatment effects from rehabilitation [21, 35]. National Institute for Health and Care Excellence [10, 11]. Supporting stroke survivors to engage in self-management and self-managed practise, requires continued support from, and good relationships between, professionals and stroke survivors characterised by trust, rapport building and emotional investment to help build stroke survivors’ confidence, resilience and sense of agency for self-management [20, 21, 36].

#### Training of PRACTISE therapist

Prior to delivering PRACTISE, physiotherapists will take part in a 1-day, in-person training session, delivered by members of the PRACTISE study team. This training will cover an overview of the PRACTISE study, detailed discussion on week-by-week delivery, instruction on the online telerehabilitation platform, G-AP and approaches to support self-management. Case studies will be used to consolidate the learning. Training materials (e.g. slides) will be made available for PRACTISE Physiotherapists to refer to along with the therapist manual. Staff can also contact the study team throughout the trial if required.

### Intervention delivery

**Fig. 2:**
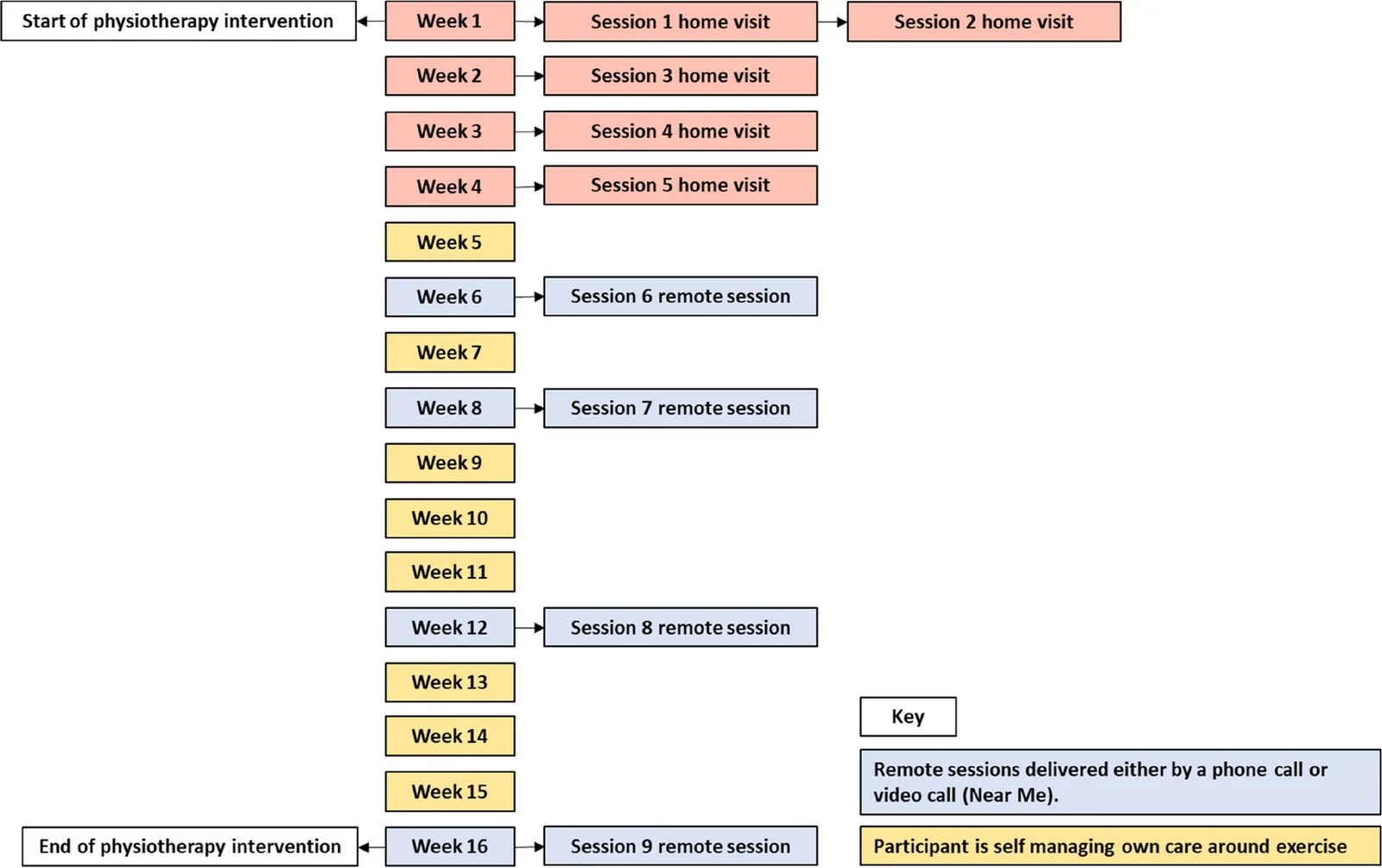
Visual representation of the structure of the PRACTISE intervention layout

The PRACTISE intervention will consist of nine sessions delivered by the PRACTISE trained Physiotherapist. The first five of these appointments will be delivered in person in the participant’s home across weeks 1–4 (Fig. 2). The remaining four appointments (weeks 6, 8, 12, and 16) will be delivered remotely via either telephone or the virtual conferencing system, Near Me (www.nearme.scot/), with the time between appointments getting longer.

During face-to-face or remote appointments, physiotherapists will review progress made by stroke survivors against their self-managed exercise practice and self-agreed, self-management action plans. These reviews will form the basis of self-practise conducted by the stroke survivor away from their physiotherapy appointments.

In weeks 5, 7, 9, 10, 11, 13, 14, and 15 participants will not have any pre-scheduled appointments with the physiotherapist. Rather, stroke survivors will be encouraged to engage in self-managed exercise practice as prescribed by the physiotherapist and related to their agreed self-management action plans. The physiotherapist will remotely review participants’ exercise diary each week, amending the prescribed exercise programme remotely as and when required.

At the start of the intervention, stroke survivors will receive a PRACTISE workbook, which has been co-developed with people with lived experience of stroke. The PRACTISE workbook will guide stroke survivors through the intervention. The workbook comprises a background to the study, a schedule of appointments, reading and reflection activities focused on goal setting and transferring the effects of practise into their daily lives through supported self-management to optimise longer term benefits. It will also include documents to help stroke survivors with the creation of their goals and associated action plans and to write notes around challenges and success they have experienced.

PRACTISE trained physiotherapy staff will also be issued with a handbook which has been developed in conjunction with NHS neuro-physiotherapists. This handbook covers the overview of the study, instructions on the use of the online rehabilitation platform, instructions and guidance on G-AP, transfer of practise, supported self-management approaches and week-by-week programme. The PRACTISE training and workbook incorporates guidance for practitioners on collaborative conversations to ensure that there is space, and time within the intervention for nurturing relationships and opportunities for stroke survivors to feel safe and supported in articulating their needs and preferences for their self-management.

In session 1 (Fig. 2), the physiotherapist will complete their clinical assessment of the stroke survivor and agree goals and action plans (G-AP) that are important to the participant. The PRACTISE Physiotherapist will have access to the Canadian Occupational Performance Measure (COPM) that has been completed as part of the baseline assessment (see below). This will allow discussion between the intervention participant and Physiotherapist to agree specific, and meaningful goals using the G-AP framework. Via self-management/G-AP approach, participants will establish specific, manageable, achievable, relevant and time-bound action plans in their pursuit of their goals.

The stroke survivor’s subsequent physiotherapy programme will be based on these goals and the physiotherapist’s assessment. The participant will have a personalised exercise programme set up on the online exercise platform where they will be asked to complete their exercises as prescribed and to record the exercises completed in the online diary within the platform. All with the aim of trying to increase rehabilitation therapy time towards the recommended guidelines. Those who may have difficulties with these tasks (e.g. because of communication impairment) will be encouraged to involve a family member/carer. In addition to the exercise component the stroke survivor will be directed towards reading specific sections within their workbook before the next session.

Subsequent appointments will follow a similar pattern, i.e. (1) the physiotherapist visits the participant at home or conducts a remote appointment; (2) a clinical review is conducted, and any relevant physiotherapy treatment is undertaken; (3) exercise diary, goals and action plans and supported self-management activities are collaboratively reviewed, discussed and adjusted as indicated, to optimise motivation and ongoing practise.

Between sessions with the Physiotherapist, participants will be asked to undertake their personalised exercise programme, up to 5 days/week depending on their ability. As the intervention progresses, participants will be supported to develop the confidence necessary to continue their own rehabilitation, maintaining optimum levels of motivation and engagement. The participant workbook will enable participants to write down their goals, plans and progress and support goal review.

PRACTISE Physiotherapist are advised to follow the PRACTISE framework as described. However, if a physiotherapist deems there is a clinical reason to change the session this will be noted and recorded as a deviation of protocol. Anticipated areas of deviation from the protocol may include the following: a physiotherapist deems the transition from face-to-face appointments to remote appointment is clinically not appropriate; a patient being discharged from care early because there is no clinical reason to continue care; and a patient needing to continue receiving care beyond the 16 weeks of the study intervention.

### IT support

For intervention participants who lack access or skills to use a suitable device to access Giraffe, the study has partnered with the charity AbilityNet (https://abilitynet.org.uk/). AbilityNet will provide support to participants, one-to-one in their home as required, to support them to access and engage with the digital content. In addition, the study will provide internet-enabled tablet computers and data packages to those who do not have access to a device. Stroke survivors and physiotherapists will also receive training and support in the use of Near Me if they have never used the system before.

### Control group

Stroke survivors randomised to the control group will receive usual physiotherapy care. Usual NHS physiotherapy is not standardised due to the nature of the condition. However, details of the content and number of treatments given will be recorded (described below).

### Both groups

Participants in both the control and intervention groups will receive usual care from other members of multidisciplinary rehabilitation team (e.g. occupational therapists, speech and language therapists).

PRACTISE is an exercise-based intervention. Any non-exercise-related physiotherapy e.g. injection for shoulder joint pain, is usual care and will follow the process and procedures within the relevant NHS board.

### Study contamination

In order to prevent study contamination, the following approaches will be in place. Physiotherapists trained to deliver PRACTISE will be asked not to treat any control participants (and vice versa). However, if, due to staffing issues this is not possible, the study team will be made aware, and it will be noted as a deviation to protocol. Stroke survivors will be reminded by the Physiotherapist not to inform the Blinded Assessor of their group allocation. If the Blinded Assessor becomes aware of study participant group allocation it will be noted down and taken into consideration for analysis.

## Data collection

Data will be recorded and collected at different stages of the intervention and are described in detail below.

## Outcomes

The outcomes of this feasibility study are (1) feasibility including process evaluation interviews (2) clinical outcomes, and (3) health economics outcomes. The results will help inform decisions on the development of a definitive clinical trial.

### Feasibility

Data from participant screening (i.e. the number of stroke survivors who are referred into community stroke services), and recruitment (those eligible who consent to the study), and follow-up logs will be used to generate realistic estimates of eligibility, recruitment, and consent. Screening will be conducted on stroke survivors who are inpatients and are being referred onto community stroke services. If stroke survivors are not referred onto this service (e.g. discharged home with no further care needed), they would not be recorded within screening logs. Screening logs will be maintained by clinical staff members within the acute, inpatient NHS facility.

Attendance and adherence for intervention participants only will be recorded to provide estimates of engagement levels and dropout. Attendance will be recorded via the number of intervention sessions completed. Adherence will be recorded as the average percentage of the weekly *unsupervised* exercise rehabilitation sessions participants complete as recommended within the national guidelines (e.g. rehabilitation at least 5 days/week). This will be measured via the exercise diary within the Giraffe platform. Data quality completeness will be assessed on the primary clinical outcome measure (Canadian Occupational Performance Measure). That is, a minimum of 80% completion rate of the COPM across all 4 time points (start, mid-point, end, and follow-up).

Fidelity of the delivery of the intervention will be measured, with stroke survivors’ and physiotherapists’ consent. Audio recordings of 20 intervention appointments will be conducted. An encrypted digital recorder will be placed in the room (face-to-face appointments) or with the treating therapist (remote sessions) to record the content of the appointment. A checklist developed using the self-management and GA-P theories that drive the intervention will be developed and applied to the transcribed recordings. Purposive, maximum variation sampling will aim for variance in week of delivery (1–16); stroke severity and delivering practitioner.

Adverse events (AE) or serious adverse events (SAE) will be assessed by any individual who has contact with the participant (e.g. treating physiotherapist, Blinded Assessor). Any AE or SAE will be reported back to the study co-ordinator for recording and onward reporting. AEs will be defined as ‘any untoward medical event affecting a study participant’ (UK Research Ethics Committees, RES SOPs V7.7, 2025). A medical event is defined as an occurrence that is beyond what would be expected following a stroke or physiotherapy exercise, is non-responsive to simple measures (e.g. rest), impacts activities of daily living, and requires consultation with a primary care healthcare professional (e.g. a general practitioner).

Although AEs are considered unlikely, potential events related to participation may include falls or near-falls (injurious or non-injurious), musculoskeletal symptoms such as muscle soreness, shoulder pain or other joint pain, other medical events beyond what would normally be expected after stroke or physiotherapy exercise that do not respond to simple measures and impact activities of daily living, fatigue exceeding typical post-stroke levels, or dizziness and syncope.

SAEs will be defined as events that result in death, are life-threatening, require hospitalisation or prolongation of existing hospitalisation, result in persistent or significant disability or incapacity, or are otherwise considered medically significant by the investigator (UK Research Ethics Committees, RES SOPs V7.7, 2025).

Any AEs and SAEs will be recorded from the time a stroke survivor consents to join the study until the last study follow-up. Clinical staff, blinded assessors or any member of the study team who interacts with the stroke survivor across the period of the study intervention will enquire about any AEs and SAEs that have occurred since the previous appointment. Stroke survivors may also contact clinical staff or members of the study team between appointments and self-report symptoms that could be classed as an adverse event. All AEs and SAEs will be recorded and reported in line with the health research authority safety report process [37].

Table 2 provides a detailed description of progression criteria to inform the development of a definitive randomised controlled trial, following guidance from previous feasibility studies that included both physiotherapy and telerehabilitation [38, 39, 40].

**Table 2:** Progression criteria for a future definitive trial

Variable	Criteria (%) (n)			Notes
	Red (stop)	Amber (modify)	Green (go)	
Initial consent	< 10%	11–19%	> 20%	Eligible participants who fit the inclusion criteria and go onto to consent to study. This is calculated as a percentage of eligible cohort that are approached and subsequently consent to study. This would be monitored regularly (e.g. monthly) in conjunction with recruiting staff to identify any barriers or facilitators that have been identified
Recruitment (*n* = 60)	< 20% *N* = < 12	21–79% *N* = 13—47	> 80% *N* = >48	Total study recruitment, across four sites
Attendance at scheduled session sessions (intervention participants only *N* = 30)	< 20% *N* = < 6	21–79% *N* = 7—23	> 80% *N* = >24	There are nine sessions within the PRACTISE intervention protocol. Seven out of nine session (77%) attended is used as the progression criteria per person. Therefore, for progression to be green on attendance for total intervention cohort we would expect to see 80% (24 out of 30) of intervention participants attend at least 77% (*n* = 7 out of 9) of their allocated appointments
Adherence to unsupervised exercise sessions (intervention participants only *N* = 30)	< 15%	16–39%	> 40%	On average what percentage of the weekly unsupervised exercise sessions do participants complete. Reporting to highlight those who completed 100% of exercise sessions will be reported to provide context
Data quality completeness	< 20%	21–79%	> 80%	A minimum of 80% completion rate of primary outcome measure (Canadian Occupational Performance Measure) across all 4 time points (start, mid-point, end, and follow-up) and as a total in both control and intervention participants
Intervention fidelity	< 20%	21–59%	> 60%	Fidelity is measured via audio recording of 20 appointments across intervention participants. Sessions will be assessed against pre-determined checklist of expected delivery. It is expected that at least 60% of items are delivered within the checklist across 20 audio-recordings

### Process evaluation interviews

Process evaluation interviews will be conducted to assess fidelity, acceptability of and adherence to the intervention. Interviews will be conducted with (1) 14 stroke survivors (10 intervention, 4 control participants); (2) 14 family members or informal carers (10 intervention, 4 control participants); and (3) PRACTISE trained intervention physiotherapists (*n* ~ 6). Interviews will be conducted after the week 16 intervention period for individuals who have experienced a stroke and for their family members or informal carers. Purposive, maximum variation, sampling will aim for variance in age, gender, stroke severity, socio-demographic background. Therapist interviews will be conducted when their involvement in the study is complete.

All interviews will be conducted by the study co-ordinator, who will not be involved in any intervention delivery or outcome assessment. Interviews will be conducted at a location and method of the participant’s preference (e.g. in person at home for participants or by phone or video call).

Stroke survivors’ interviews will explore how they experienced the treatment they received; factors that influenced their ability to engage with treatment; factors outside the treatment that affected their experience of treatment, their experiences of self-managing their rehabilitation; and their experiences of the assessments.

Family members or informal carers’ interviews will explore factors that impacted (positively or negatively) their experience of having a significant other receiving treatment; the impact on relationships; their confidence; burden or additional support provided and their experiences of their own assessments.

Physiotherapist interviews will explore experience of delivering the PRACTISE intervention; factors (positive or negative) that influenced delivery of the core components of PRACTISE; remote delivery approaches and physiotherapists’ perceived perception of the stroke survivors’ experience.

### Clinical outcomes

All clinical outcome measures will be collected at four assessment points, except the patient experience of treatment and self-management after stroke (PETS-stroke) which will be collected at baseline and week 16 only. Most of the included tests have been recommended for stroke rehabilitation trials [41]. Clinical outcomes will be collected by a blinded assessor.

#### Blinded assessors

Blinded assessors, who are blinded to group allocation will conduct data collection assessments with stroke survivors. Assessors will undertake training that will include an overview of the PRACTISE study, study documentation and awareness and conducting of assessments. Assessors will get the opportunity to practise the administration and recording of each of the outcome measures with research team members who experienced in the delivery of the assessment. This will allow assessors to gain confidence in the delivery, furthermore, provide a level of inter-rater consistency on scoring physical assessments (e.g. Action Reach Arm Test). Training materials (e.g. slides and videos) will be made available to refer back to and the assessor can also contact the study co-ordinator when required.

**Fig. 3:**
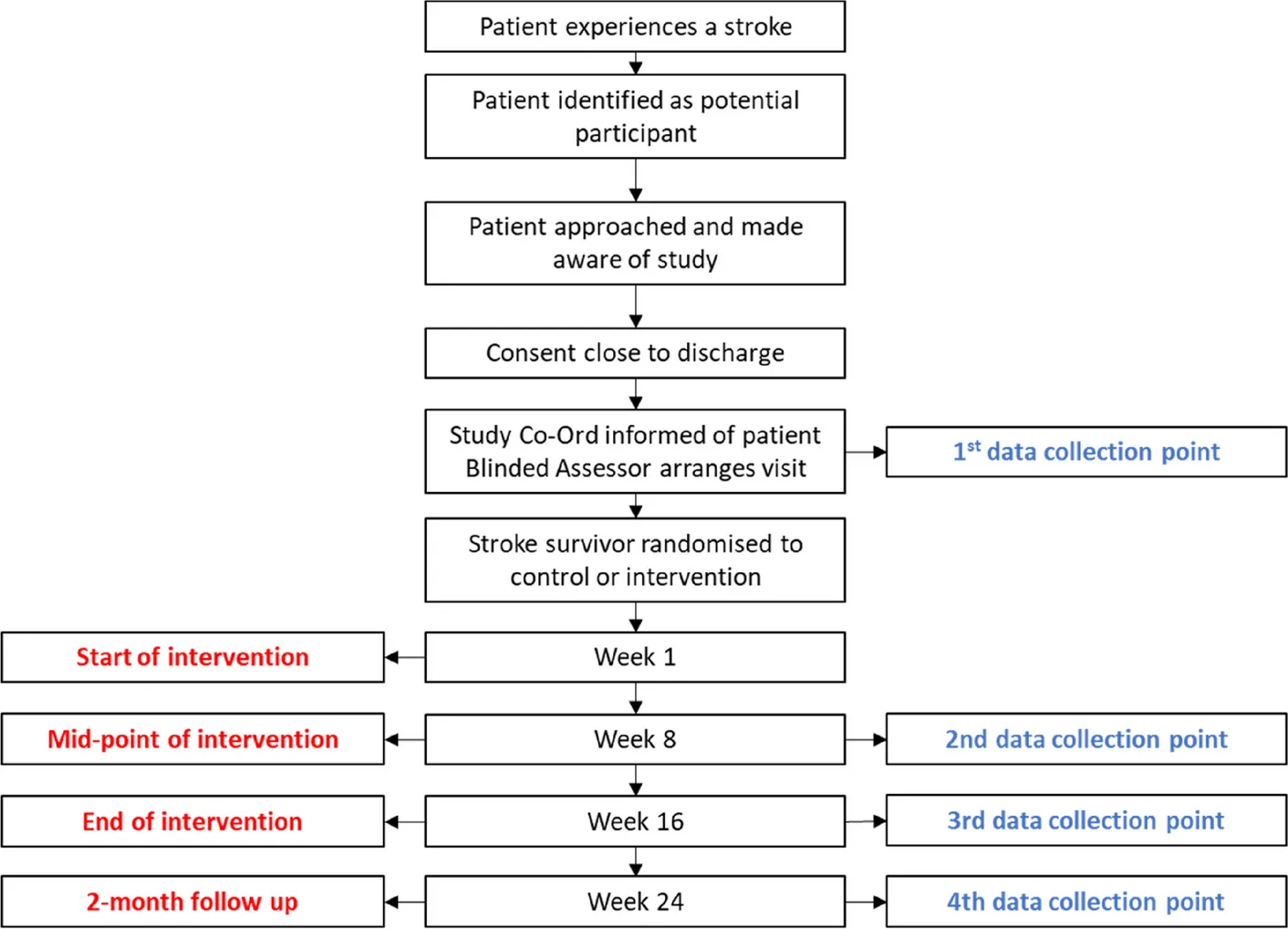
Visual representation of data collection time points in relation to study timeline

Assessors will conduct physical assessments with stroke survivors and where required, aid participants with any written assessments. Blinded Assessors will visit participants home on four occasions; at base line (prior to randomisation/the start of the intervention period), and in week 8, week 16 (end of intervention) and week 24 (2-month follow up) (Fig. 3). To reduce potential assessment burden, written assessments can be completed in a participant’s own time rather than during the assessment sessions. If participants would prefer this. Forms will be sent to stroke survivors in advance of the visit, allowing a few days for them to be completed beforehand. If the stroke survivor is unable to complete the questionnaires independently, they can be assisted by a carer/family member or the assessor during the assessment visit.

#### Demographic data

Demographics will be collected from each relevant group of participants after they have consented to the study.

#### Stroke survivors

For stroke survivors, demographics will be recorded by a Research Nurse, member of the clinical team, or study co-ordinator, when the participant is an inpatient. These will include age, sex, ethnicity, highest level of education, address and postcode (to enable identification of indices of multiple deprivation and facilitate home assessor visits). Stroke details will also be recorded, including date of stroke, stroke type, sub-type. The Charlson Comorbidity Index [42] will be used to record any co-morbidities. The Charlson Comorbidity Index is a weighted index to predict risk of death within 1 year of hospitalisation for patients with specific comorbid conditions. However, this study will not use the index as a predictor of mortality, rather as a tool for identifying co-morbidities.

Two measures, traditionally used as outcome measures, will be utilised to describe participants in terms of level of disability and cognition. The modified Rankin Scale (mRS) will be utilised in part to ensure a participant fits the inclusion criteria (mRS 2–4) for the study and to characterise their level of disability. The mRS is a single item global outcomes rating scale of functional independence which ranges from 0—no symptoms to 5—severe disability [43] and has been recommended for stroke rehabilitation trials [41, 44].

The Oxford Cognitive Screen (OCS) will also be collected and used to characterise participants’ level of cognition [45]. The OCS is a stroke-specific and domain-specific cognitive screening tool which can be delivered at the bedside in acute stroke. The OCS is easy to administer and score and importantly is inclusive for patients with aphasia and neglect [46]. The OCS domains assessed are language, praxis, number, memory, spatial and controlled attention.

#### Informal carers or family members

Demographics for family members will be collected by a research nurse, member of the clinical or research team, depending on where and who takes consent. Demographics will include age, sex, relation to the stroke survivor, employment, ethnicity, and highest level of education.

#### Treating physiotherapist

Demographics will be collected from physiotherapy staff by a member of the research team. Age, ethnicity, sex, physiotherapy grade/band, and years of experience in working in stroke rehabilitation will be recorded.

### Primary clinical outcome

The primary clinical outcome in this study is the Canadian Occupational Performance Measure (COPM). The COPM is a personalised, patient-centred, and responsive assessment, capturing meaningful changes in daily activities that matter most to stroke survivors. The COPM is designed to detect change in a patient’s self-reported performance and satisfaction over time [47], [48]. Within the COPM the participant identifies up to five problems they face in self-care, productivity and leisure. They then prioritise the activities and rate their current level of performance and satisfaction with each. Performance and satisfaction ratings for each identified problem are summed and averaged to generate mean scores out of 10 at baseline and at subsequent assessment providing an overall measure of perceived occupational performance and satisfaction [48]. A change of two or more points is commonly considered clinically significant [49], although some uncertainty persists regarding the validity of this threshold across different populations and contexts [50]. The COPM is generally a reliable, valid, and acceptable tool for researchers with satisfactory test–retest reliability specifically in stroke [51].

Full COPM results will be sent to the treating physiotherapist prior to their first appointment with the participant (intervention only) to facilitate the discussion between the stroke survivor and physiotherapist to agree specific and meaningful goals.

### Secondary clinical outcomes

All secondary outcomes have acceptable reliability and validity and are widely used in stroke studies. All secondary outcomes relate to the domains of upper limb function, upper limb function, mobility, motor impairment, activities of daily living, stroke-specific quality of life, self-efficacy, and patient experience of treatment and self-management.

The action research arm test (ARAT) is a 19-item measure of upper limb function with 4 sub-tests (grasp, grip, pinch, and gross arm movement) [52]. Each item is scored on a four-point scale from 0 (can do no part of the test) to 3 (performs the test normally). Participants are first asked to perform the most difficult task within a subscale. If a participant performs the task normally, they are scored a 3 on this item and all remaining items within that subscale. A score between 0 and 2 on the first item suggests that the second task (least difficult) must be evaluated. Participants scoring 0 on the second task item are unlikely to be successful on subsequent scale items and are scored 0 for remaining tasks within a category and are thus not evaluated further on that subscale. Otherwise, all test items within a category must be performed. Consequently, participants may be asked to perform between 4 and 19 tasks depending on their performance. Scores on the ARAT may range from 0 to 57 points, with higher scores indicating better performance.

The motricity index [53] assesses muscle strength of the upper and lower limbs and involves three upper limb movements: shoulder abduction, elbow flexion and pinch grip and three lower movements: hip flexion, knee extension and ankle dorsiflexion. The maximum score is 100 for both the upper limb and for the lower limb. These scores are then added together before being divided by 2 for a final score of 0–100 for the test. The test has excellent reliability and excellent construct and concurrent validity [54].

Timed Up and Go test assesses mobility, balance, walking ability, and fall risk [55]. The participant stands up from a chair, walks 3 m at a comfortable pace, turns around, walks back to the chair and sits down. The time to complete the test is recorded and walking aids are used as required. The test will be conducted three times with the average score being calculated and used as the final measure.

The patient-reported outcome measures (PROMS) for stroke survivors are the following:

The modified Barthel index is an outcome of degree of independence in performing 10 activities of daily living (ADL), with scoring ranging from zero to 20 [56]. The higher the score, the greater the degree of functional independence. While observation of activities is preferred, asking an informed individual close to the patient is as reliable as testing, and is quicker [56]. Therefore, to reduce burden on participant, the assessor will ask the stroke survivor or where appropriate (e.g. communication difficulties) an informal carer or family member. The modified Barthel index scale demonstrates good reliability and sensitivity to changes in ADL ability (REF).

The stroke-specific quality of life questionnaire consists of 49 items each assessed on a 5-point scale [57]. The domains covered include mobility, energy, work and productivity, mood, self-care, language, thinking and personality. Scores range from 49 to 245 with higher scores associated with higher quality of life.

EQ-5D-5L evaluates health-related quality of life with one question for each of the five dimensions: mobility, self-care, usual activities, pain/discomfort, and anxiety/depression [58, 59]. Each dimension is scored: (1) no problems, (2) slight problems, (3) moderate problems, (4) severe problems and (5) extreme problems. While a score of 11,111 indicates no problems across the five dimensions, a score 55,555 would indicate extreme problems across all dimensions. The EQ-VAS records the participant’s self-rated health on a vertical visual analogue scale where the endpoints are labelled ‘the best health you can imagine’ and ‘the worst health you can imagine’. The VAS will be used as a quantitative measure of the participants’ perception of their health with higher scores indicating better perceived health.

Fatigue Severity Scale is 9-item scale [60]. The scale measures the severity of fatigue and its effect on a person’s life against these 9 statements. Each item is scored on a scale from 1 (strongly disagree) to 7 (strongly agree). Scores range from 9 to 63 with higher scores indicating more severe fatigue.

Stroke Self-Efficacy Questionnaire (SSEQ) is a self-report scale of self-efficacy post stroke that demonstrates good internal consistency and criterion validity [61]. It is a 13-item PROM where participants rate their confidence with stated tasks from 0 (not at all confident) to 10 (very confident). The SSEQ total score can range from 0 to 130 with higher scores indicating higher levels of self-efficacy.

The patient experience of treatment and self-management after stroke (PETS-stroke) (collected at baseline and week 16 only) is a 34-item patient reported measure that captures the workload of healthcare and impact on well-being experienced by the stroke survivor [62]. PETS-stroke is comprised of nine subscales and one single item that each report on different aspects of treatment burden, with scores recorded at subscale level [63].

#### Falls

As the intended outcome of the intervention is to increase functional activity this may increase risk of falls. Falls are self-reported, with a fall defined as ‘an unexpected event in which the participant comes to rest on the ground, floor or lower’. Falls will be captured as an adverse event (described above).

#### Burden outcome measure

Recruited family members or informal carers will be asked to complete the modified caregiver strain index (CSI) [64] at each of the four assessment points. The CSI is a 13-item questionnaire which assesses the level of stress experienced by informal caregivers, answered with—yes (2), sometimes (1) or no (0). The maximum score is 26, with a higher score indicating a higher level of strain. The CSI has demonstrated acceptable reliability and construct validity, and its brevity makes it suitable for use in both clinical and research settings [64]. Family members or informal carers will be sent the measure either via post or completed when the blinded assessor is visiting their significant other, if appropriate.

#### Dose–response and adherence measures

##### Therapy checklist: both groups

Any rehabilitation delivered by members of the multidisciplinary team (e.g. physiotherapy, occupational therapy and speech and language therapy) will also be recorded by the relevant clinical staff member at each appointment they deliver for both control and intervention participants. Data recorded will include date, duration and location of appointment (e.g. clinic, telerehabilitation), nature of the appointment (e.g. speech and language therapy) and travel time to appointment (if a home visit). In addition, physiotherapists and occupational therapists will also complete a checklist of interventions delivered during the appointment (e.g. balance–standing activity, co-ordination activity–upper limb). This data will provide some detail of usual care.

##### Exercise adherence

Exercise diaries will be completed by stroke survivors either within the Giraffe platform (intervention) or on paper (control). Treating therapists will remind stroke survivors to complete their diaries. This information will be used, in conjunction with the therapy checklist, to determine the weekly dose of exercise sessions adhered to, from which dose–response will be calculated. Understanding the preliminary dose–response relationships and the extent to which exercise dosage influences recovery outcomes is central to refining the content and intensity of a definitive study.

## Health economics outcomes

The economic analysis will focus on the feasibility and acceptability of data collection for conducting an economic evaluation in a future trial. This will involve self-reported healthcare resource utilisation, personal costs (Stroke survivors and family or informal carers), intervention costs, and health-related quality of life (HRQoL) via the EuroQol 5D-5L tool. The EuroQol 5D-5L will be collected at baseline, weeks 8, 16, and 24 as described above from stroke survivors. All stroke survivors will be asked to document any resources accessed and purchased across the 16-week intervention via a paper form. This will include any planned and/or unplanned healthcare contacts in the last week (e.g. services accessed, frequency, and if relates to their stroke), any new medication to treat their stroke or stroke symptoms (prescribed and over the counter), and any equipment purchased for stroke-related rehabilitation. Travel time to attend face-to-face physiotherapy appointments, associated costs (e.g. parking fees), and carer/companion time spent assisting with rehabilitation (including attending appointments) will also be recorded. Data on intervention device use (device type, e.g. smart phone) and access (internet connection costs) will be collected for participants in the intervention arm.

Participants will be asked to record any difficulties accessing or any assistance required from informal carers/friends when using the online platform. We will also explore the feasibility of collecting data informing the environmental impact of an online versus in-person rehabilitation intervention in this context (e.g. carbon footprint of streamed rehabilitation versus travel to clinic). Data are anticipated to be rudimentary and drawn from participant-reported resource utilisation.

## Analysis

The statistical analysis and reporting of the feasibility outcomes will be descriptive in nature. Analysis of the process evaluation interviews will follow the Framework Analysis Approach [65] and will be managed within an appropriate platform (e.g. Nvivo). Analysis will be informed by Normalisation Process Theory [66]. Specifically, interviews will be interpreted through NPT components of coherence, cognitive participation, collective action, reflective monitoring to deepen understanding of implementation for future evaluation [67].

Analysis of the clinical outcomes will focus on the feasibility and acceptability of data collection for a future trial. This will be predominantly descriptive in nature. The COPM will additionally be analysed in the following way. The 24-week COPM data and estimate the between-group mean from an ANCOVA model adjusting for baseline COPM and design covariates. To gain extra precision, we will analyse the COPM using repeated-measures mixed-effects regression model correcting for baseline score and time as a categorical fixed effect and centre, and participant as a random effect. The repeated measures will be assessed at week 8 and 16 and week 24 (follow-up) with treatment effect, which is the difference in means at each time point, estimated from the time-by-treatment interaction, presented with 95% confidence intervals. Where appropriate, the remaining clinical outcomes will be analysed in a similar manner using a generalised linear model suitable for the distribution of the outcome. Consistent with the feasibility design, no formal hypothesis tests will be undertaken, and p-values will not be reported.

Costs will be attached to healthcare resource use using standardised unit costs where available. Market values for year of data collection will be applied to the costs of the online intervention, over the counter medication, and equipment purchases. Aggregated and disaggregated costs will be descriptively compared between intervention arms. The feasibility of estimating carbon emissions from participant-reported travel times (where private transport was used to access clinical services) and device use will be reported and presented where available data allows.

## Patient and public involvement and trial steering committee

A patient and public involvement (PPI) group of six stroke survivors has been formed and will meet five times over the course of the study. They will serve as advisory partners throughout the study, contributing to the development of the research approach, addressing challenges from a PPI perspective, informing the interpretation of findings in context, and supporting the dissemination of results upon study completion. Two members from the PPI group will also sit on the Trial Steering Committee (TSC) and represent the PPI group. Those involved in PPI activities have been, and will continue to be, reimbursed in line with INVOLVE guidelines [68]. To date, the PPI group has provided feedback on the stroke survivors’ workbook, outcome measure layout and presentation, and helped in identifying potential hurdles and facilitators to intervention delivery.

The Trial Steering Committee (TSC) includes four external clinicians and researchers with experience in stroke rehabilitation alongside the two members of the PPI group. The TSC, who will meet five times over the study duration, will provide independent oversight, ensure the study adheres to ethical and scientific standards, and support the integrity and safety of the trial as it progresses.

## Dissemination

The study will provide updates to national governing bodies or charities for stroke such as the Stroke Association, Different Strokes and Chest Heart and Stroke Scotland. We will use social media platforms (e.g. LinkedIn) associated with organisations such as Stroke Association, and Chest Heart and Stroke Scotland, Different Strokes and national stroke voices to share appropriate updates and information. We will also work with the Stroke Association to produce aphasia friendly reports. We will host a hybrid engagement event in Glasgow Caledonian University for stroke survivors and their families, who will be able to join in person or online. We will also look to share findings through clinical networks to ensure stroke therapists and other relevant healthcare professionals are aware of the findings. Findings will also be shared via relevant academic conferences such as the UK Stroke Forum, European Stroke Organisation Conference, Scottish Stroke Care Audit and CPD events such as the National Stroke Sessions.

We will publish academic papers in open access journals and the papers will also be publicly available in Glasgow Caledonian University research repository PURE (www.gcu.ac.uk/currentstudents/essentials/library/research/pure). Data will be deposited in an external repository, OSF (https://osf.io/), which allows researchers to host structured datasets and associated documentation. Datasets in OSF receive a DOI which we will reference in publications other dissemination activities.

## Discussion

Current UK national clinical guidelines (2023) recommend that stroke patients should receive at least 3 h/day of multidisciplinary rehabilitation at least 5 days/week. However, workforce shortages, increasing service demands, and variability in access to community-based rehabilitation hamper the ability to achieve this level of rehabilitation. Therefore, effective, innovative and scalable models of rehabilitation that can supplement standard care and support sustained recovery beyond hospital discharge are required.

This feasibility study has been designed to generate the necessary evidence to inform the potential progression to a definitive trial. The use of stop–go criteria is central to this process. Criteria, developed a priori, will provide explicit thresholds to guide decisions regarding whether, and in what form, a full-scale RCT is justified. The inclusion of an embedded process evaluation, guided by the Medical Research Council’s updated framework for complex interventions [69] will help explore essential insights into the mechanisms of action, contextual influences, and implementation challenges associated with the intervention. Specifically, qualitative and quantitative data will help to identify areas where the intervention requires refinement, whether in content (e.g. tailoring of goal setting strategies), delivery (e.g. the role of telerehabilitation), or support for self-management. From the therapist perspective, the process evaluation will seek to highlight potential facilitators and barriers to fidelity, workload implications, and training needs. From the stroke survivor’s perspective, it will shed light on issues of engagement, acceptability, and perceived relevance of the intervention to their rehabilitation journey.

A TiDieR framework will be produced with the result from this feasibility study. This will then provide a framework for the definitive trial to ensure transparent reporting and to systematically document intervention delivery. In doing so, we will seek to generate an understanding of how and why the intervention may work in practise, thereby offering opportunities to develop and refine an emerging programme theory exploring how telerehabilitation, goal setting, and self-management can be integrated to optimise post stroke recovery.

## Data Availability

Data will be deposited in an external repository; OSF (https://osf.io/), which allows researchers to host structured datasets and associated documentation. Datasets in OSF receive a DOI which we will reference in publications other dissemination activities.

## Abbreviations

AE: Adverse event
ARAT: Action Research Arm Test
COPM: Canadian Occupational Performance Measure
G-AP: Goal Setting and Action Planning Framework
mRS: Modified Rankin Scale
OCS: Oxford Cognitive Screen
PETS-Stroke: The Patient Experience of Treatment and Self-Management After Stroke Questionnaire
PIS: Participant Information Sheet
PPI: Patient and Public Involvement
PRACTISE: Personalised Community Based Telerehabilitation Post Stroke to Increase Rehabilitation Time and Improve Motor Recovery
RCT: Randomised controlled trial
SAE: Serious adverse event
SSEQ: Stroke Self Efficacy Questionnaire
TiDieR: Template for Intervention Description and Replication
TSC: Trial Steering Committee
TUG: Timed Up and Go Test
